# Enhanced electrical conductivity and stretchability of ionic-liquid PEDOT:PSS air-cathodes for aluminium-air batteries with long lifetime and high specific energy

**DOI:** 10.1038/s41598-022-26546-8

**Published:** 2022-12-21

**Authors:** Hatim Machrafi, Fabio Iermano, Souhail Temsamani, Ilija Bobinac, Carlo S. Iorio

**Affiliations:** 1 Physical Chemistry Group, Université libre de Bruxelles, Brussels, Belgium; 2 GIGA-In Silico Medicine, Université de Liège, Liège, Belgium; 3 UFR Physique, Sorbonne Université, Paris, France; 4 Politecnico Di Milano, Milan, Italy

**Keywords:** Characterization and analytical techniques, Design, synthesis and processing, Batteries

## Abstract

A hydrogel film, poly-3,4-ethylenedioxythiophene (PEDOT):polystyrenesulfonate (PSS), containing an ionic liquid, is used as an air–cathode for a metal-air battery and its performance is investigated. This work presents the development of the air–cathode and the characterization of its physical, chemical and mechanical properties. Moreover, in view of wearable batteries, these air-cathodes are implemented within a flexible aluminium-air battery. It contains an aluminium anode, an electrolyte made of cellulose paper imbibed with an aqueous sodium chloride solution and the PEDOT:PSS air–cathode. Characterisation tests showed that the ionic liquid did not change the air–cathode chemically, while the electric conductivity increased considerably. The anode has an acceptable purity and was found to be resistant against self-corrosion. Discharge tests showed operating voltages up to 0.65 V, whereas two batteries in series could deliver up to 1.3 V at a current density of 0.9 mA cm^−2^ for almost a day, sufficient for monitoring and medical devices. Several discharge tests with current densities from 0.25 up to 2.5 mA cm^−2^ have presented operating lifetimes from 10 h up until over a day. At a current density of 2.8 mA cm^−2^, the operating voltage and lifetime dropped considerably, explained by approaching the limiting current density of about 3 mA cm^−2^, as evidenced by linear sweep voltammetry. The batteries showed high specific energies up to about 3140 Wh kg^−1^. Mechanical tests revealed a sufficient stretchability of the air–cathode, even after battery discharge, implying an acceptable degree of wearability. Together with the reusability of the air–cathode, the battery is a promising route towards a low-cost viable way for wearable power supply for monitoring medical devices with long lifetimes and high specific energies. Optimization of the air–cathode could even lead to higher power applications.

## Introduction

Research and industry have put a great deal of effort into the development, elaboration and production of new types of batteries with better performance and with specific properties^1^. Among these, a growing interest has been developed in bendable batteries. Indeed, such batteries are seen to be at the heart of the development of portable and flexible electronic systems, renewable energy storage as well as biomedical technologies requiring a source of energy that can adapt and take various forms^2^. Indeed, the proliferation of wearable electronic products has highlighted the need for a technological adaptation by the power sources. As a result, the batteries which were generally used in applications where their rigidity and their volume were of little importance, are no longer suitable for wearable technologies which will favor their compactness and their flexibility. As an example, we can cite the development of smart watches, flexible smartphones as well as sensors, medical dressings for health monitoring (where dressings could need power supply to provide information on the state of health) and Internet of Things^2,3^. In addition to presenting a certain practical aspect, such batteries could make it possible to obtain storage systems with a much higher capacity despite a limited volume. Indeed, developing an electrochemical cell with a small thickness and with the ability to bend could lead to the formation of a battery with a large capacity by folding it back on itself. Therefore, the bendability of a battery could also influence its performance and in particular its capacity. When it comes to applications for which a restricted weight is expected, precisely of importance for wearable energy sources, special attention will be given to the specific energy and power. One of the batteries that have high specific energies are of the metal-air type, as air acts as the cathode catalysed by a porous medium, gaining more attention^4–7^.

**Figure 1:**
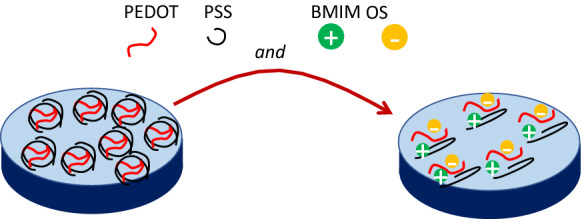
Schematic representation of molecular interaction between the IL BMIM:OS and the PEDOT:PSS, where the PEDOT:PSS is decoupled into PSS^−^ and PEDOT^+^ with the ions BMIM^+^ and OS^-^. Large-scale conducting domains are thus created.

The electrode is an important element for such batteries. The air cathode deserves special attention in metal-air batteries. Standard cathodes used in these batteries are typically Platinum (Pt) catalysts and alloys but suffer from cost-ineffeciency and scarcity^8^. Although not sufficiently sustainable, we might also mention other used materials: metal oxides, transition-metal macrocycles or nitrogen-doped carbon-based materials^9^. Having advantages in being chemical stable, processed at low temperatures, flexible and biocompatible, recent attention has been given to conductive polymers (CPs). The discovery that polyacetylene (PA) could achieve high conductivity for an organic molecule resulted into an increase of interest in CPs^10^. Nonetheless, in its regular state it showed semiconducting behavior (conductivity $\sim {10}^{-7}$ S m^−1^), as most other conjugated polymers show^11^. The major advantage of conductive polymers is their higher processability^12,13^. However, they may suffer from poor durability affecting cycle performance, although this can be remediated by adding, for instance, carbon fillers, such as carbon nanotubes or graphene^14^. Other advanced techniques for dispersion and doping can also enhance their electrical properties. As an example, iodine vapors are known to enhance the conductivity of polyacetylene to the order of metals^15,16^. Such doping was responsible in increasing the electrical conductivity from ${10}^{-7}$ S m^−1^ up to ${10}^{5}$ S m^−1^, where it should be noted that the conductivity of copper is around ${10}^{8}$ S m^−1^^3^. These doping procedures allowed the use of a series of different conducting polymers, such as polyaniline, polypyrrole and polytiophenes, to mention a few. Polyaniline has high stability and processability but looses conductivity at neutral or high pH^17,18^. Polypyrrole has the advantage of forming easily composites from it, while it suffers from thermal degradation due to the loss of dopants^17,19^. Polytiophenes gained great interest due to their environmental stability, thermal stability and high optical properties in comparison to other conducting polymers^17^. From these polythiophenes, we mention poly-3,4-ethylene dioxythiophene (PEDOT) as a mature material that is under development and one of the most successful ones^17,20–24^. The electric conductivity of PEDOT is of the order $O({10}^{2})$ S/m^25^. Its main problem, however, is its insolutiblity in water, which can be overcome by introducing readily available counter-ions, such as polystyrene sulfonate (PSS), into a polyelectrolyte complex (PEC)^17^. Fully stable PEDOT:PSS can be produced by oxidative chemical polymerization of EDOT in the presence of PSS, where Na_2_S_2_O_8_ is used as an oxidative agent^26,27^. This procedure results into a PEDOT:PSS hydrogel, which can swell when brought into contact with aqueous solutions. This property opens several applications for electrodes, membranes, sensors or batteries. Secondary doping is able to further improve the electric conductivity. An interesting way of doing so is by adding chemical doping agents, by means of a solution-based procedure, resulting into a hydrogel with higher stability and conductivity. A typical doping agent is an ionic liquid (IL). ILs are organic cation/inorganic anion salts, having melting temperatures typically below 100 °C. The reason behind using ILs for the enhancement of the electrical conductivity of CPs stems from the charge screening effect of the ILs, which cause a higher interconnection and crystallinity of PEDOT nanofibrillar structures^28^. It is believed that the IL components help the PEDOT:PSS to decouple into PSS^−^ and PEDOT^+^, allowing them to grow into large-scale domains with increased conductivity^28,29^. The principle is a general one and several ILs are used in the literature, such as ethyl-3-methylimidazolium (EMIM):tetracyanoborate (TCB), EMIM:tricyanomethanide (TCM), EMIM:ethylene sulfate (ES) and EMIM:chloride (Cl)^30,31^. Another IL is 1-butyl-3-methylimidazolium (BMIM):octyl sulfate (OS), which is not only used for its capability to enhance considerably the electric conductivity of PEDOT:PSS, but also for increasing its stretchability with respect to many others^32^. Figure 1 shows schematically how on a molecular level the IL intervenes with the PEDOT:PSS. In this case, the IL BMIM:OS is shown.

The usual way to dope PEDOT:PSS with ILs is by mixing aqueous PEDOT:PSS solutions with ILs and sonicating them^30^. In this work, a solution-based preparation of an IL-doped PEDOT:PSS cathode is presented, followed by a physical, chemical and mechanical characterization to assess its usefulness within the framework of low-cost wearable metal-air batteries. The IL chosen is 1-butyl-3-methylimidazolium:octyl sulfate, due to its high enhancement of the electrical conductivity and stretchability, important for applications concerning wearable electrodes, as mentioned earlier. As for the metal anode, we propose to use low-cost commercial aluminium foil, one of the most abundant elements in earth’s crust. The Methods section presents the used materials, battery assembly, its working principle and the used characterisation techniques. In fine, the development and characterization of an aluminium-air low-cost flexible battery are treated in this paper as a viable way towards wearable power sources.

## Results

### Characterisation of the PEDOT:PSS cathode

**Figure 2:**
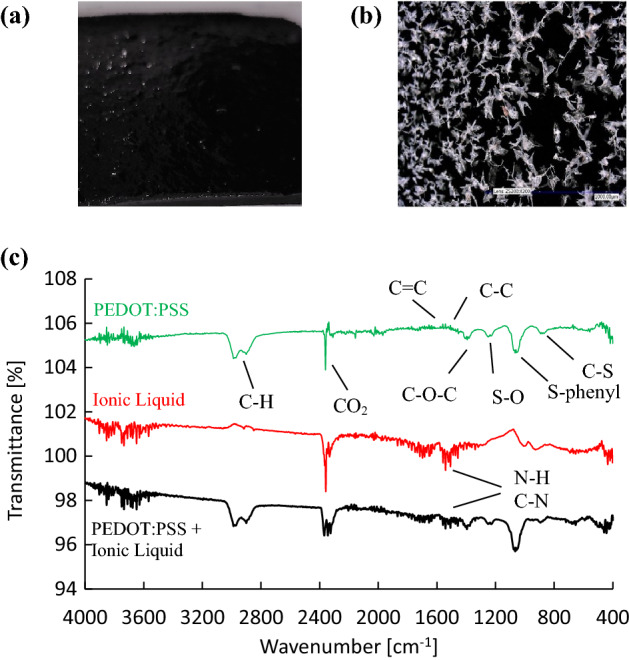
(**a**) PEDOT:PSS cathode, (**b**) SEM image of PEDOT:PSS film with 200 × magnification. (**c**) FTIR profiles of pure PEDOT:PSS, pure IL and IL-doped PEDOT:PSS, where typical band spectra are indicated and where it should be noted that the spectra are shifted (vertically) for better visualization.

Images of the electrodes are shown in Fig. 2. Figure 2(a) shows an image of a PEDOT:PSS electrode, obtaining a smooth, flexible and stable sheet, while Fig. 2(b) shows a SEM image with a 200 × magnification of a PEDOT:PSS electrode.

Figure 2(b) shows a black background with white spots. Performed EDS analysis has shown that the black background contains mainly sulfur and oxygen elements indicating the PEDOT:PSS hydrogel. The EDS analysis of the white crystals reveals, besides a higher presence of oxygen, also sodium. As during the preparation of PEDOT:PSS a use was made of Na_2_S_2_O_8_ as an oxidative agent^26,27^, the white crystals are most probably composed of this oxidative agent.

The resistivity and profilometry measurements are given in Table 1 for the pure and two ionic-liquid-doped PEDOT:PSS electrodes with two thicknesses.

**Table 1:** Electric performance of pure and two ionic-liquid-doped (ILD) PEDOT:PSS electrodes.

PEDOT:PSS electrode	${R}_{s}$[$\Omega$]	$\delta$[$\mu m$]	$\varrho$[m $\Omega m$]	$\sigma$[kS/$m$]
Pure	180	52	9.3	0.11 ±0.01
ILD1	0.36	109	0.039	25.6 ±3
ILD2	0.57	65	0.037	27.0 ±3

The electric conductivity of pure PEDOT:PSS is of the same order of magnitude as other reported values in^3,25^. It can be seen from Table 1 that the ionic liquid increases indeed the electric conductivity of the PEDOT:PSS electrode considerably by two orders of magnitude. It seems that the relatively simple procedure of preparing the IL-doped electrodes is quite reliable as it gives the same order of magnitude for the film thickness and, more importantly, the electric conductivity. The stability of the chemical composition can also be shown by comparing the FTIR analyses of the pure PEDOT:PSS electrode to the IL-doped one in Fig. 2(c). The PEDOT:PSS FTIR spectrum shows C = C, C–C, C–O–C, S–O, S-phenyl and C–S stretching bands at wavenumbers between 800 cm^−1^ and 1600 cm^−1^^33^ and C-H stretching bands around 2950 cm^−1^^34^. These bands are also found in the IL-doped PEDOT:PSS, in addition to supplementary bands of larger intensity between 1500 cm^−1^ and 1600 cm^−1^. The FTIR spectrum of the IL shows that these bands come from the IL, indicating C–N stretching and N–H bending bands^35^. Note that the peaks around 2350 cm^-1^ indicate the absorption of CO_2_^36^, most probably background CO_2_. As the FTIR spectrum of IL-doped PEDOT:PSS does not show significant band shifts due the IL (only additional bands are observed at the same wavenumbers as found for the pure IL) in comparison to pure PEDOT:PSS, this suggests that the chemical composition of the electrode is unchanged and only the electrical conductivity is enhanced.

We note that we prepared also IL-doped PEDOT:PSS electrodes adding up to 5 mol% of CuCl_2_ as it is reported to enhance the electrical conductivity at high content^37^. However, we noticed that at high contents the PEDOT:PSS lost its flexibility, which is one of the properties sought after in this work. Therefore, we had to limit the addition of CuCl_2_ to the IL-doped PEDOT:PSS electrode up to 5 mol%, which appeared to have no significant influence on the electrical conductivity. The effect of CuCl_2_ on the electrical conductivity of the IL-doped PEDOT:PSS electrode is therefore discarded within the framework of this study.

### Self-corrosion resistance of the aluminium anode

**Figure 3:**
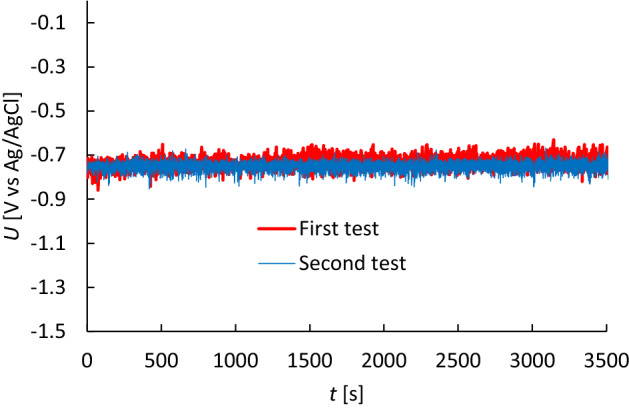
Open circuit potential test of the aluminium anode. Two tests of two aluminium foil samples are performed for reproducibility.

As for the anode, XRF measurements showed a 98.5% purity for the element aluminium, the remaining impurities containing mainly iron, silicon and oxygen. Concerning the open circuit potential (OCP) testings that were performed to study self-corrosion of the aluminium, a 2 M aqueous sodium chloride NaCl electrolyte was used. Figure 3 shows OCP measurements of two aluminium foil anodes before use.

Figure 3 shows a stable OCP value. This indicates that there is no significant corrosion due to the aqueous NaCl electrolyte. Another work studied specifically the corrosion evolution of aluminium in NaCl solutions and reported that no corrosion was observed^38^. These results suggest that for the purposes of this work the commercial aluminium anode is deemed to be inert for self-corrosion in the presence of the NaCl electrolyte.

### Battery discharge testing

The maximum operating voltage of a battery is defined as the operating circuit voltage (OCV) ${E}_{cell}$, related to the theoretical standard potential (TSP) ${E}_{cell}^{\ominus }$ via the Nernst equation:

1 $$E_{cell} = E_{cath} - E_{an} = E_{cath}^{{ \ominus }} - E_{an}^{{ \ominus }} - \frac{RT}{{zF}}\left( {\ln \frac{{{\mathfrak{a}}_{R,cath} }}{{{\mathfrak{a}}_{O,cath} }} - \ln \frac{{{\mathfrak{a}}_{R,an} }}{{{\mathfrak{a}}_{O,an} }}} \right) = E_{cell}^{{ \ominus }} - \frac{RT}{{zF}}\ln Q_{r} ,$$

where, the subscripts “$\mathrm{cath}$” and “$\mathrm{an}$” denote the properties related to the cathode and anode half-cell reactions, respectively, while ${\mathcal{a}}_{R}$ and ${\mathcal{a}}_{O}$ stand for the activities of the reduced and oxidized forms, respectively, of the relevant species in the half-cell reactions and ${Q}_{r}$ the overall reaction quotient of the cell reaction. Equation (1) has been written in a general form, where it should be noted that the activity of solid reactants is usually taken as unity and that of gaseous reactions is usually expressed in terms of a gas pressure. This equation shows that if the activities of all the constituents would be equal to unity (i.e. ${Q}_{r}=1$), the OCV would be the same as the TSP. In practice, the OCV is even less than what the Nernst equation would predict. For aluminium-air batteries, mean operating voltages between 0.4 and 0.8 V are reported, depending on the electrolyte: alkaline electrolytes showing mean operating voltages closer to 0.8 V and neutral electroltyes rather in the range 0.4 to 0.6 V^39,40^. However, batteries with alkaline electrolytes show higher self-corrosion of the anode reducing the life time of the battery with respect to those with neutral electrolytes via the reaction $2Al+6{H}_{2}O\stackrel{{OH}^{-}}{\to }2Al{\left(OH\right)}_{3}+3{H}_{2}$^39,40^. Operating voltages are also observed to suffer from higher current densities^41,42^. The preference of the electrolyte resides into the application. For applications where high voltages or high current densities are not needed but the focus is more on the duration (several hours or days), such as for many wearable medical and monitoring devices^43–45^, neutral electrolytes are preferred: as no self-corrosion occurs no additives are needed, keeping the cost low. Furthermore, as we are dealing with low-cost and light-weight batteries, should higher voltages be still needed, it suffices to use 2 or 3 batteries in series, as long as the specific energy is high.

**Figure 4:**
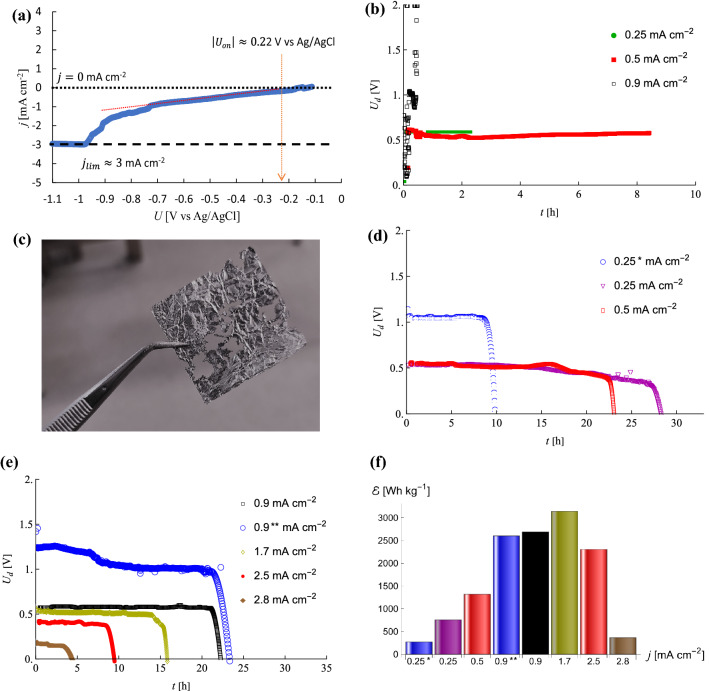
(**a**) LSV curve of the PEDOT:PSS cathode indicating the limiting current for the ORR and the onset potential for oxygen reduction by the catalyst (**b**) Discharge curves of Al-air batteries with golden current collector for different current densities (**c**) Partially consumed aluminium anode (**d**) Discharge curves of Al-air batteries for different low current densities, where, * indicates a non-optimal battery assembly of two batteries in series (**e**) Discharge curves of Al-air batteries with moderate to higher (approaching the limiting current) current densities, where, ** indicates two batteries in series (**f**) Specific energy for several current densities.

The PEDOT:PSS air–cathode, acting as a catalyst for the oxygen reduction reaction (ORR) ${O}_{2}+2{H}_{2}O+4{e}^{-}\to 4{OH}^{-}$, has undergone a linear sweep voltammetry (LSV) in order to appreciate its electrochemical properties. Figure 4(a) shows an LSV result where it can be seen that the current has a plateau value at potentials under − 1 V vs Ag/AgCl. This current is called the limiting current ${j}_{lim}\approx 3$ mA cm^−2^ and shows that beyond this current oxygen diffusion through the catalyst will be limiting the ORR to occur properly. It is then also expected that higher current densities will not result into the functioning of a battery with this catalyst as the air cathode. On the other hand, the absolute value (as the working electrode in the LSV setup acts as the cathode, the voltages are negative) of the onset potential seems to be around 0.22 V versus Ag/AgCl, which seems to indicate a relatively low kinetic barrier for the ORR to occur. This may be the consequence of an improved quality of the electric conductivity of the air cathode as discussed earlier. During the discharge of the battery, whilst the oxygen is reduced at the air cathode, the reaction taking place at the anode is given by $Al+3{OH}^{-}\to Al{\left(OH\right)}_{3}+3{e}^{-}$. Figure 4(b) presents discharge voltages ${U}_{d}$ as a function of time $t$ at different constant discharge current densities $j$, using as a current collector thin golden paper. During the assembly of the battery, this current collector appeared to be prone to cause contact problems because it shifted during the handling of the battery. Therefore, we can see in Fig. 4(b) that the discharge curves are not completed (even though attaining up to about 8 h), resulting into an incomplete consumption of the aluminium anode (see Fig. 4c) and a poor specific energy. Moreover, we observed also at the end a sharp increase in the potential, indicating short circuit behavior. For this reason, we switched to a more solid copper current collector (noting that other forms of golden current collectors could be considered). We assembled two batteries in series with a copper current collector and measured the discharge curve, presented in Fig. 4(d), at a constant current density of 0.25 mA cm^−2^. Altthough the discharge curve presents a normal tendency, its lifetime is quite short. The reason for this was found to be that upon assembling the batteries and tightening from the sides, the middle section appeared to be in poor contact. This was remediated by assuring a tight contact in the middle section as well, where, in order to assure the adequacy of the assembly, we limited the rest of the discharge tests (except for one, as illustration) to one battery assembly at a time. At the same current density, i.e. at 0.25 mA cm^−2^, the life time was for the improved assembly longer than a day. This type of battery assembly was then tested by varying the current densities of which the results are presented in Fig. 4(d,e). It can be seen that the discharge curves show voltages between 0.55 and 0.65 V (within the expected voltage for aluminium-air batteries with neutral electrolytes and no additives, as mentioned earlier) and long lifetimes, ranging from 15 h to more than a day for current densities ranging from 1.7 to 0.25 mA cm^−2^, respectively. Figure 4(e) shows also that for a current density of 2.5 mA cm^−2^, the lifetime was still 10 h, with a power output of the order of 1 mW cm^−2^, largely sufficient for biocompatible batteries^43–45^. At a current density of 2.8 mA cm^−2^, however, the discharge operating voltage becomes unacceptably low. This is understood by reminding that the measured limiting current was about 3 mA cm^−2^ in Fig. 4(a). As an illustration, Fig. 4(e) also shows the discharge curve of two batteries in series at a current density of 0.9 mA cm^−2^. It shows for the initial 6 h an operating voltage around 1.3 V, whereafter it drops steadily towards around 1.1 V up to over 20 h. Such a series shows that long discharge times can be achieved with such low-cost, easy-to-assemble batteries with operating voltages that are of the order of magnitude of batteries that use alkaline electrolytes, albeit that the present ones show higher lifetimes.

### Specific power evaluation

Figure 4(d,e) showed a relatively constant useful voltage from 10 h to more than a day, which highlights its ability to supply an electrical current and make it usable for applications. It is interesting to calculate the energy deployed by the batteries, where we are only interested in the ones presented in Fig. 4(d,e). The energy can be calculated from integrating the power $P$ over time until complete discharge after time ${\tau }_{d}$. The power equals the discharge potential ${U}_{d}$ times the current ${I}_{d}={A}_{b}j$, where ${A}_{b}$ is the surface of the electrodes through which the current passes, $j$ is the current density and ${U}_{d}(t)$ is given by the discharge curves. The battery energy is then given by

2 $$E_{d} = \int_{0}^{{\tau_{d} }} {Pdt} = \int_{0}^{{\tau_{d} }} {U_{d} I_{d} dt}$$

The specific energy $\upepsilon$ is obtained by dividing ${E}_{d}$ by the mass of the aluminium anode and is presented in Fig. 4(f) for various current densities. It shows that the battery with the poor current collector connection (with a current density of 0.25 mA cm^−2^ indicated by an asterisk) has a relatively low specific energy. The specific energy (at the same current density) with an improved connection shows an almost triple value. As the current density increases, the specific energy increases as well until around 3140 Wh kg^−1^ at a current density of 1.7 mA cm^−2^. This is a high value and motivates the possibility of a useful aluminium-air battery for light-weight low-cost long-duration applications. Upon approaching the limiting current, the specific energy decreases to around 2300 Wh kg^−1^, being still quite high though until dropping under 400 Wh kg^-1^ at a current density of 2.8 mA cm^−2^. As the presented aluminium-air battery has a low cost and little weight, the operating voltage, when needed, may be increased by putting the batteries in series. We can see in Fig. 4(f) that the specific energy of two batteries in series at a current density of 0.9 mA cm^−2^ (indicated by two asterisks) is around 2600 Wh kg^−1^. This is quite close to the specific energy of one battery at the same current density, i.e. around 2690 Wh kg^−1^, but the series of two presenting an operating voltage up to 1.3 V, as is shown in Fig. 4(e). In addition, but for the purposes of this work, more importantly, the life time of the battery remains quite high, i.e. almost one day.

### Assessment of cathode flexibility

**Figure 5:**
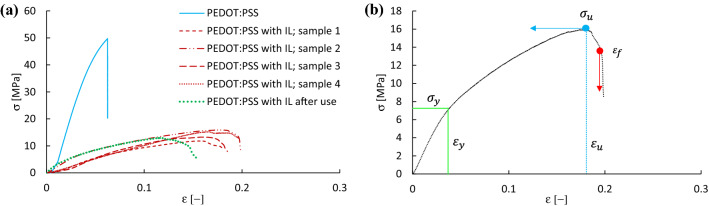
(**a**) Stress–strain curves for the PEDOT:PSS films with and without IL doping and a measurement of an IL-doped PEDOT:PSS after its use as a cathode during a battery discharge. (**b**) Schematic representation of the stress and strain definitions for Table 2.

It is interesting to assess the stretchability of the IL-doped PEDOT:PSS electrodes. For this reason, we performed tensile strength measurements for the electrodes, comparing them to a PEDOT:PSS film before IL doping in stress–strain curves in Fig. 5(a). Four IL-doped PEDOT:PSS electrodes were tested, using the same fabrication method, to assess reproducibility of the level of stretchability, named sample 1 to 4, respectively. First, Fig. 5(a) shows that, globally, after doping the PEDOT:PSS film with the ionic liquid the stress in the elastic regime reduces considerably with respect to the pure PEDOT:PSS film, indicating an improved elasticity due to the ionic liquid. It appears also that after the PEDOT:PSS film has been used as a cathode during battery discharge the stress–strain relationship is quite similar to that before its use, implying a preserved elasticity. Further information can be extracted from Fig. 5(a). From the yield stress ${\sigma }_{y}$ and the strain at yield ${\varepsilon }_{y}$ in the elastic region, we can obtain Young’s modulus ${E}_{y}\equiv \frac{{\sigma }_{y} }{{\varepsilon }_{y}}$, assuming a solely linear elastic region up to ${\varepsilon }_{y}$, a possibly existing non-linear region being difficult to extract from Fig. 5(a). The maximum stress is called the ultimate tensile strength ${\sigma }_{u}$, at an ultimate tensile strain ${\varepsilon }_{u}$, followed by the strain at fracture ${\varepsilon }_{f}$. The latter two values do not enter into the discussion of stretchable elastic films, where we assume returning to the initial shape after unloading. However, they are interesting values to tabulate as they are still an indication of the strength of the polymeric network of the films. These definitions are illustrated in Fig. 5(b) using one of the curves as an example. Table 2 shows the extracted values.

**Table 2:** Stress and strain values of the PEDOT:PSS films, extracted from Fig. 4(a).

PEDOT:PSS films	${\sigma }_{y}$[MPa]	${\varepsilon }_{y}$[%]	${E}_{y}$[GPa]	${\sigma }_{u}$[MPa]	${\varepsilon }_{u}$[%]	${\varepsilon }_{f}$[%]
No IL	19	1.6	1.2	49	6	6
With IL–1	4.4	3.2	0.14	11	14	17
With IL–2	5.5	2.6	0.21	16	18	20
With IL–3	5.0	3.3	0.15	13	17	18
With IL–4	4.6	4.4	0.10	15	17	18
After use	3.7	1.4	0.26	13	11	15

It should first be noted that, although two significant numbers are given in Table 2, the linear elastic regions in the stress–strain curves are not clearly discernable, which allows a qualitative assessment of the mechanic properties of the PEDOT:PSS films and quantitatively we can only make conclusions on the order of magnitudes. Having this in mind, Table 2 shows that the ionic liquid, as already globally observed in Fig. 5(a), indeed increases considerably the elasticity of the PEDOT:PSS films by decreasing 5 to 6 times its Young’s modulus from about $1$ GPa to about $2\times {10}^{2}$ MPa. The ultimate tensile strain and fracture strain for the film without IL are equal, indicating a neat break, in contrast to the films with IL where a maximum ultimate tensile stress is followed by a decrease in the stress before the film breaks at a slightly higher strain. These values imply that fracture would occur after between 10 and 20% strain, which is an indication of the maximum stretch of a battery to avoid a short circuit with the current collectors.

## Discussion

The primary aim of this research project was to propose a method to produce a solution-based preparation of hydrogel cathodes for the purposes of a viable aluminium-air battery in view of powering monitoring devices for medical and/or wearable sensing. The cathode was an ionic-liquid doped PEDOT:PSS hydrogel and the anode came from a commercial aluminium foil. In a metal-polymer battery, in addition to acting as a catalyst for metal-air reactions, the polymer cathode can undergo changes during the discharge process^46^. In particular, it has been proven that oxidation–reduction reactions take place on the surface of the polymer. Previous cyclic voltammetry tests^3^ have, however, shown that these reactions were reversible and that therefore the PEDOT:PSS cathode was reusable. As the anode is made from commercial aluminium foil, this results into a flexible low-cost battery.

Physical and mechanical characterisations have shown that the ionic liquid increased considerably the electric conductivity without altering the stretchability of the PEDOT:PSS films. Chemical analyses showed that the preparation method results into a reliable reproducibility of the hydrogel films as well as a negligible self-corrosion of the aluminium anode. These characterisation tests indicate a sufficiently stable, stretchable and conductive films to be used as cathode in an aluminium-air battery. Discharge curves at a constant 1 mA current showed that the battery delivered the current for more than 8 h, the limit being determined by the evaporation of the electrolyte base fluid. Otherwise, it would have lasted even longer. Such a problem could be overcome by developing a different configuration that assures a better tightness of the setup, whilst allowing contact with air, or by using aerogel or solid electrolytes, for instance. Nevertheless, the results suggest promising applications for aluminium-air batteries with PEDOT:PSS as the cathode as power supply for monitoring medical devices. Although aluminium is used as the metal, this work could be applied to other metal-air batteries in general. Improvements can also be made concerning the external battery holders or even considering different fabrication methods, such as printing on thin films. For applications where higher power outputs are necessary, one may imagine stacking several of these batteries while keeping a reasonable weight and high degree of wearability.

Although the stress–strain curve of the cathode after use looks similar to that before battery discharge, the Young’s modulus after use seems to increase somewhat, indicating a slight increase of stiffness after battery discharge. It is interesting to know whether this has any consequences on the stretchability of the IL-doped PEDOT:PSS films in realistic configurations. Table 2 showed that the elastic region seems to be delimited by a stretching of approximately 3% before its use as a cathode and about half of it after battery discharge. We can make a qualitative assessment whether this stretchability is enough for the use in a wearable flexible battery. Let us consider a film that we bend in a shape corresponding to a hemi-sphere, where the bottom side has a length corresponding to the initial one $L$, and the top side has a length that corresponds to a stretched length $L+\Delta L$. Geometric considerations show that $\Delta L=\pi \delta$, with $\delta$ the film’s thickness. With a typical thickness around 0.1 mm, we would have a maximum stretch of about 0.3 mm. The films used for the tensile strength measurements had a length around 15 mm. A strain of 1.5 to 3% would correspond to a stretch of about 0.2 to 0.4 mm. Most of the applications for wearable batteries would not attain hemi-spheric bending shapes, but most probable with curvatures corresponding to bending angles of much less than 90°. This means that the stretch would most probably be significantly less than 0.3 mm. Thinner films and higher elasticities could improve even more the stretchability of these IL-doped PEDOT:PSS films, whilst bending and twisting tests should also be performed next to performing battery discharge tests in different rotational and angular positions of the battery in order to assure a full wearability. It is to be noted that the battery contained commercial aluminium for the anode and just cellulose filter paper for the electrolyte-containing separator. In addition, the cathode being reusable and stretchable, while aluminium being the most abundant metal in Earth’s crust, the proposed battery not only provides for a low-cost bendable version of metal-air batteries, but can push the cost even lower with respect to the use of other metals as the anode. Moreover, aluminium-air batteries have higher theoretical specific power densities than other metal-air batteries^47^. The aluminium-air batteries used in this work have shown high specific energies, around 3140 Wh kg^−1^ whilst presenting low-cost and easy assembly. The electrocatalyst for the oxygen reduction reaction is shown to have a low kinetic barrier but it can still be improved to enhance the oxygen reduction and allow higher current densities. The present analysis has demonstrated that the stretchability of these films, when used as a cathode in a bended metal-air battery, would be reasonably maintained throughout the battery discharge, indicating a realistic feasibility of using IL-doped PEDOT:PSS hydrogels as metal-air battery cathodes.

## Methods

### Materials

The preparation of the cathode is a solution-base procedure. The main ingredient for the electrode preparation consists of an aqueous PEDOT:PSS dispersion CLEVIOS™ PH 1000, purchased from Heraeus, with a solid content between 1.0 and 1.3%. The PEDOT:PSS electrode consists of using ionic salts as both stabilizers and electric enhancers. The ionic salt used for enhanced electric conductivity is 1-butyl-3-methylimidazolium octyl sulfate (ionic liquid, Sigma Aldrich, ≥ 96% HPLC). Additions of anhydrous copper(II) chloride (CuCl_2_, Alfa Aesar, min 98%) are reported to have little effect at low content, but may increase electrical conductivity when added to PEDOT:PSS at high content^37^. However, we already chose to use an ionic salt because it not only increases the electrical conductivity but serves in addition for stability enhancement. Therefore, we only make a short assessment of the addition of CuCl_2_ to PEDOT:PSS and don’t investigate this any further. More information can be found in^3^. The electrolyte used for the battery device is a 2 M aqueous sodium chloride (NaCl) solution. The separator for the battery is a Whatman® cellulose chromatography 1 Chr paper, purchased from Sigma-Aldrich.

### Ionic-liquid-based PEDOT:PSS electrode preparation

A sonicated aqueous PEDOT:PSS dispersion is mixed with the ionic liquid 1-butyl-3-methylimidazolium octyl sulfate. After mixing, 5 g of the mixture was poured into a 25 cm^2^ square and 5 mm deep Teflon mold. A mold is used in order to maintain a regular thickness. Drying lasted for 18 h at room conditions. Annealing was done in a preheated oven at 130 °C for 25 min inspired from the procedure described in^28^. The ionic liquid content has been set to 60 wt% of the final product. For the assessment of the effect of CuCl_2_ on an ionic-liquid-based PEDOT:PSS electrode, the ionic liquid content was fixed at 45 wt% and adding to it CuCl_2_ content up to 5 mol%. The samples are took out from their molds before they are used as a cathode for the battery.

### Battery assembly

**Figure 6:**
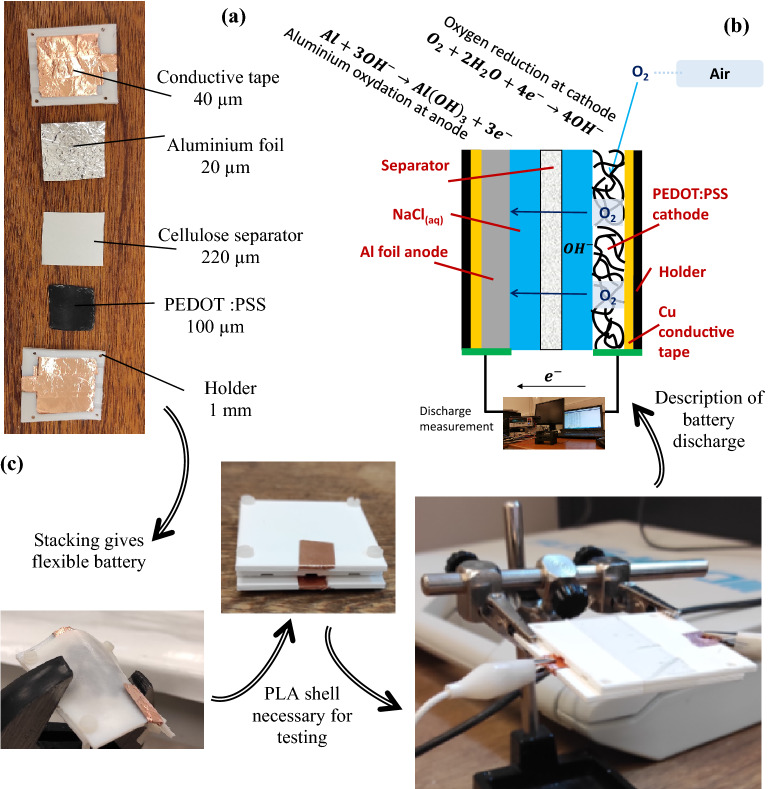
(**a**) Battery components, (**b**) schematic representation of the battery functioning and the chemical reactions during battery discharge, (**c**) assembled flexible battery and discharge testing.

Figure 6 presents the components used for the assembly, the assembly procedure and a schematic description of the battery discharge. A current is measured, induced by the oxygen reduction at the cathode and the oxidation reaction at the anode. To perform the discharge measurement correctly, the battery was held between polylactic acid (PLA) support sheets. A particular geometry with a groove for the battery elements and holes to allow assembly was designed with the SolidWorks software^48^ and produced with the Ultimaker 3 3D printer. The grooves designed have a thickness of 0.4 mm, which ensure good adhesion of the various components once assembled, allow the necessary passage of air and provides for terminals that can be connected to the measuring instruments. The doped PEDOT:PSS hydrogel electrode serves as the cathode, while a 20 µm thick commercial aluminium foil serves as an anode. Cellulose filter paper, with pore sizes from 20 to 25 µm and a thickness of 220 µm, separates the electrodes. To ensure a good connection, a 40 µm thick copper foil (golden paper was also considered) was glued directly to the PLA supports and pressed, respectively, on both sides of the electrodes. Before montage, the separator was imbibed with the aqueous NaCl solution. Finally, all these components were stacked together and then everything was held with Teflon screws. After the assembly of the sensor, it was connected to a Keithley 2400 SourceMeter SMU measuring instrument, and the discharge test was started at a constant controlled operating current.

Figure 6 also shows the chemical process that takes place during the discharge. The imbibed separator has also the function to make the PEDOT:PSS hydrogel swell by contact with water, creating micro/nanopores for oxygen to diffuse through and enabling the oxygen reduction reaction. As such, the electric circuit is closed, and a redox reaction starts to occur. The oxygen from the air is reduced at the PEDOT:PSS cathode, releasing hydroxyl ions that diffuse through the electrode towards the aluminium anode where it oxydizes aluminium to aluminium-hydroxide ^4^. The reaction occurring at the PEDOT:PSS cathode is represented by ${\mathrm{O}}_{2}+2{\mathrm{H}}_{2}\mathrm{O}+4{\mathrm{e}}^{-}\to 4{\mathrm{OH}}^{-}$, whereas at the Al anode the reaction $\mathrm{Al}+3{\mathrm{OH}}^{-}\to \mathrm{Al}{(\mathrm{OH})}_{3}+3{\mathrm{e}}^{-}$ occurs. It is easy to see that the total reaction is $4\mathrm{Al}+3{\mathrm{O}}_{2}+6{\mathrm{H}}_{2}\mathrm{O}\to 4\mathrm{Al}{(\mathrm{OH})}_{3}$. Although not subject of this work, it should be noted that there are side reactions that can cause corrosion at the anode^4,49^.

### Characterization methods

Four-probe resistance measurements, FTIR, Profilometry, X-ray fluorescence spectroscopy, open circuit potential and tensile strength measurements have been used to characterize the electrodes.

### Four Probe resistivity measurement

The primary technique for measuring sheet resistance is the four-probe method (also known as the Kelvin technique). The electrodes must transport electrical charge laterally and need low sheet resistances to reduce losses during this process. Furthermore, the resistivity and conductivity can be calculated if the sheet resistance and material thickness are known. This allows for the materials to be electrically characterized, purely by measuring their surface resistivity. A four-probe system consists of four electrical probes in a line, with equal spacing between each of the probes. The measurement of surface resistance with this four-probe system operates by applying a current $\mathrm{I}$ on the outer two probes and measuring the resultant voltage drop $\mathrm{V}$ between the inner two probes. For a uniform spacing $\mathrm{S}=7$ mm between the probes (with contact diameter of 0.4 mm), and a sheet thickness much smaller than the probe spacing, the sheet resistance is given by ${\mathrm{R}}_{\mathrm{s}}=\mathrm{C}\frac{\uppi }{\mathrm{ln}2}\frac{\mathrm{V}}{\mathrm{I}}$^50^, where we introduced a geometrical correction factor $\mathrm{C}$. The geometrical correction factor equals unity when the sample dimensions (length $\mathrm{L}$ and width $\mathrm{W}$) are significantly larger than the probe spacing, i.e. $\mathrm{L}\gg \mathrm{S}$ and $\mathrm{W}\gg \mathrm{S}$ so that the sheet resistace is ${\mathrm{R}}_{\mathrm{s}}\equiv {\mathrm{R}}_{\mathrm{s}}^{\infty }$. By varying the length and width of the samples, measuring the sheet resistance and comparing it to ${\mathrm{R}}_{\mathrm{s}}^{\infty }$, a calibration test is performed. Knowing the electrode sample width and length, it can be interpolated that $\mathrm{C}\approx 0.81$. As the sheet resistance is not a material property, it is more appropriate to speak in terms of the sheet resistivity $\mathrm{\varrho }$ given by $\mathrm{\varrho }={\mathrm{R}}_{\mathrm{s}}\updelta$, where $\updelta$ is the sample thickness. The sample thickness is obtained by means of profilometry and digital microscopy. The surface electric conductivity is simply given by $\upsigma ={\mathrm{\varrho }}^{-1}$.

### Profilometry

A Veeco Dektak® 150 Surface Profiler has been used to measure the sample thickness of the cathode. The profiler measures the surface topography by converting the vertical movement of a stylus in contact with the sample surface into an electrical signal^51^.

### FTIR Analyses

FTIR analyzes have been performed on the PEDOT:PSS electrodes to detect the chemical bonding and changes at the surface of the structures. The samples were analyzed with a Jasco FT/IR-6600 FT-IR Spectrometer.

### SEM and EDS analyses

A Hitachi SU-70 scanning electron microscope was used with accelerating voltage of 20 kV. Non-conductive samples were treated with gold using a low-vacuum sputter deposition. After interaction with primary electrons X-ray photons are generated. These are formed as a side effect of electron transition from a higher excited state to a lower one^52,53^. Signals created in this way could be analyzed in order to determine the chemical composition of materials, since every element will create its own distinct X-ray spectra. For this analysis, Energy Dispersive X-ray spectroscopy (EDS)^52^ is performed using the Hitachi SU-70 machine.

### X-ray fluorescence

The X-ray Fluorescence (XRF) spectroscopy is a non destructive characterization technique used to quantitatively determine the elements that compose a tested sample. In the framework of this work, XRF testings were performed by a Bruker S4 PIONEER in order to analyze the relative purity of the aluminium anode.

### Open circuit potential measurements

In order to determine the self-corrosion rate of the aluminum anode in a cell that is not in a working mode (i.e. when no current is produced), it was necessary to reproduce those specific conditions into an experimental procedure. Thereby, it was decided to perform an Open Circuit Potential (OCP) testing: into an electrochemical device containing three electrodes immersed in an electrolyte, we form a specific electric circuit that would be connected to a potentiostat in order to measure potential variation as a function of time. Those three electrodes are referred as working electrode, counter electrode and reference electrode. The working electrode is the one to be studied, i.e. the anode. The counter electrode is placed to complete the circuit while the reference electrode is used as a potential reference.

### Tensile strength measurements

A force–displacement measurement were performed of the cathode samples to test their stretchability. For this, we used a Shimadzu AGS-X tensile testing apparatus. The sample is placed between an upper and a lower holder. Then, at a rate of 1 mm/min, the sample is stretched until it breaks. The necessary force and displacement are measured, and, knowing the dimensions of the samples, converted into stress–strain curves.

### Discharge test

In order to characterize the electrical performances of the batteries, several discharge tests were performed on the battery. This consists in connecting the battery to a sourcemeter device that allows the measurement of the electric potential for a given electric current in order to obtain discharge curves. As it happens it permitted to get the discharge voltage ${\mathrm{U}}_{\mathrm{d}}$ versus time and for an imposed discharge current ${\mathrm{I}}_{\mathrm{d}}$ that was of 1 mA. The choice of the discharge current allows observing the process for the longest period of time. The discharge testings were performed at stabilized ambient room temperatur. The testing was performed using the Keithley 2400 Sourcemeter SMU.

### Linear sweep voltammetry

The performance of the air cathode to catalyse the oxygen reduction reaction is assessed by means of linear sweep voltammetry (LSV). We used a pre-printed mini three-electrode system on which the PEDOT:PSS electrode was printed with a Dimatix Materials Printer DMP-2850 to act as a working electrode, together with a carbon counter electrode and a Ag/AgCl reference electrode. A NaCl solution has been used as the electrolyte. The pre-printed cell was connected to the μStat400 bipotentiostat/ galvanostat measuring instrument and immersed in a beaker containing the electrolytic solution. The working electrode potential is ramped linearly versus time, measuring the current density.

## Data Availability

The datasets used and/or analysed during the current study are available from the corresponding author on reasonable request.
